# Utilizing 3D Point Cloud Technology with Deep Learning for Automated Measurement and Analysis of Dairy Cows

**DOI:** 10.3390/s24030987

**Published:** 2024-02-02

**Authors:** Jae Gu Lee, Seung Soo Lee, Mahboob Alam, Sang Min Lee, Ha-Seung Seong, Mi Na Park, Seungkyu Han, Hoang-Phong Nguyen, Min Ki Baek, Anh Tuan Phan, Chang Gwon Dang, Duc Toan Nguyen

**Affiliations:** 1 National Institute of Animal Science, Rural Development Administration, Cheonan 31000, Chungcheongnam-do, Republic of Korea; 2 ZOOTOS Co., Ltd., R&D Center, Anyang 14118, Gyeonggi-do, Republic of Korea

**Keywords:** dairy cow, point cloud registration, automatic measurement and analysis, machine learning, deep learning

## Abstract

This paper introduces an approach to the automated measurement and analysis of dairy cows using 3D point cloud technology. The integration of advanced sensing techniques enables the collection of non-intrusive, precise data, facilitating comprehensive monitoring of key parameters related to the health, well-being, and productivity of dairy cows. The proposed system employs 3D imaging sensors to capture detailed information about various parts of dairy cows, generating accurate, high-resolution point clouds. A robust automated algorithm has been developed to process these point clouds and extract relevant metrics such as dairy cow stature height, rump width, rump angle, and front teat length. Based on the measured data combined with expert assessments of dairy cows, the quality indices of dairy cows are automatically evaluated and extracted. By leveraging this technology, dairy farmers can gain real-time insights into the health status of individual cows and the overall herd. Additionally, the automated analysis facilitates efficient management practices and optimizes feeding strategies and resource allocation. The results of field trials and validation studies demonstrate the effectiveness and reliability of the automated 3D point cloud approach in dairy farm environments. The errors between manually measured values of dairy cow height, rump angle, and front teat length, and those calculated by the auto-measurement algorithm were within 0.7 cm, with no observed exceedance of errors in comparison to manual measurements. This research contributes to the burgeoning field of precision livestock farming, offering a technological solution that not only enhances productivity but also aligns with contemporary standards for sustainable and ethical animal husbandry practices.

## 1. Introduction

Modern agriculture is experiencing a transformative evolution, with advancements in sensor technologies playing a pivotal role in reshaping traditional farming practices. In the domain of livestock management, particularly in dairy farming, the integration of cutting-edge technologies offers unprecedented opportunities for optimizing productivity, ensuring animal welfare, and promoting sustainable practices. This paper introduces a pioneering approach to the automated measurement and analysis of dairy cows, leveraging the capabilities of 3D point cloud technology. The convergence of precision livestock farming and sensor-based methodologies promises a paradigm shift in how farmers monitor and manage their dairy herds.

Dairy farming, a cornerstone of the global agricultural landscape, confronts multifaceted challenges spanning productivity and resource optimization to animal health and welfare. Traditional methods of monitoring cows often rely on manual measurements and subjective observations, which can be time-consuming, labor-intensive, and prone to human error. In response to these challenges, the proposed system harnesses the power of 3D point cloud technology, offering a non-intrusive, accurate, and automated means of capturing detailed anatomical information.

The utilization of 3D imaging sensors enables the generation of high-resolution point clouds that provide a comprehensive representation of the physical characteristics of dairy cows. These point clouds serve as a rich source of data for the development of sophisticated algorithms aimed at extracting valuable metrics related to body dimensions, posture, and health indicators. By automating the measurement and analysis processes, the proposed system not only alleviates the burden on farmers but also opens new avenues for in-depth and continuous monitoring, enabling timely interventions and preventive measures.

**Figure 1:**
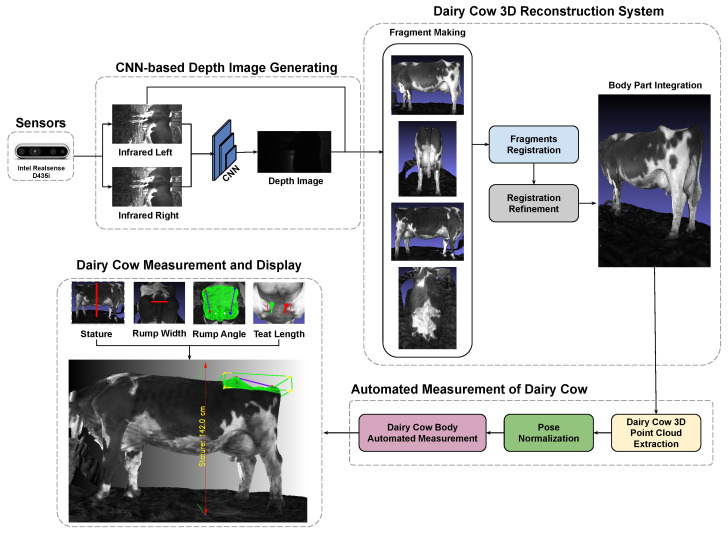
The automated measurement and analysis of dairy cows framework.

The significance of this research extends beyond the realm of traditional livestock management. As the agricultural landscape embraces smart farming practices, the integration of cloud-based platforms for data storage and analysis becomes imperative. The scalability and accessibility afforded by cloud technologies empower farmers to remotely access real-time insights into the health and well-being of their dairy herds. This paper unfolds the conceptual framework, methodology, and potential implications of the automated 3D point cloud approach, representing a promising stride toward a technologically enriched and ethically sound future for dairy farming, detailed as shown in Figure 1.

Particularly, the contribution of this paper can be summarized as follows:

1. The multi-camera synchronization system helps minimize outliers and increase the accuracy of dairy cow 3D reconstruction, thereby avoiding factors that seriously affect the accuracy of dairy cow body size measurement.
2. Enhancements to the previous 3D reconstruction system result in more precise stitching between the bottom and top cameras based on the camera system’s initialization matrix.
3. Automatic measurement and analysis of various parts of dairy cows are conducted with high accuracy.

The remainder of this paper is organized as follows: Section 2 reviews the automated measurement and analysis processes algorithm and their applications in dairy farming tasks. In Section 3, a reconstruction framework for improving the dairy cow 3D point cloud quality is presented. In Section 4, the automated measurement and analysis of dairy cows via a 3D point cloud approach is proposed. Section 5 presents the experimental results and the evaluations of the proposed approach in the multiple datasets. Section 6 concludes this paper.

## 2. Related Works

The use of 3D imaging technology has found applications in diverse fields, including agriculture. Researchers have investigated its potential in crop monitoring, yield prediction, and now in livestock management. The ability of 3D imaging to provide detailed spatial information makes it a promising tool for capturing and analyzing the three-dimensional structure of dairy cows, presenting opportunities for accurate measurement and assessment.

Automated systems for monitoring livestock have evolved significantly, moving beyond traditional manual methods. Computer vision and machine learning techniques have been employed to automate the recognition of animal behaviors and health conditions. These studies often utilize 2D imaging systems, and the incorporation of 3D point cloud technology represents a novel extension to enhance the granularity and accuracy of data collection.

In accordance with [1], the authors implemented a method for automatically extracting measurements to estimate the weight of Nellore cattle based on regression algorithms using 2D images of the dorsal area. Additionally, the use of depth images along with an algorithm for automatically estimating heifer height and body mass for cattle, as presented in [2], has demonstrated that in single-view measurement methods utilizing a single RGB camera or depth camera for body condition and body size characteristics evaluation, challenges persist in obtaining multi-scale information, such as chest girth, abdominal circumference, rump angle, and so on.

Similar approaches for single-view based measurement problems have also been discussed in [3,4,5,6,7]. For the task of dairy cow 3D reconstruction, the farm environment and the movement of dairy cows significantly impact the resulting point cloud generated using multi-view methods, leading to the appearance of outliers and distortion in the dairy cow 3D point cloud. Consequently, the evaluation of body size introduces considerable errors [8]. By constructing a synchronized multi-camera system [9] during the point cloud generation process, our current approach has successfully minimized the occurrence of outliers, and the visualization of the dairy cow point cloud has been significantly improved.

On the other hand, the measurement of dairy cows is divided into two levels: manual and automatic measurement. The manual measurement method requires identifying measurement points on images and point clouds. Subsequently, the body-side parameters are calculated by determining the distance between the marked points. Automated measurement is achieved through the manual or automatic filtering of input images or point clouds [10,11], which is followed by the automated measurement of animal body size.

## 3. The Dairy Cows 3D Reconstruction

Currently, to successfully reconstruct a dairy cow, it is crucial to ensure the proper conditions on the farm, an effective data collection system, and the state of the dairy cow at the time of implementation. The excessive movement of dairy cows during the data collection process for 3D reconstruction is a significant concern due to the adverse effects it introduces. Specifically, the RGBD-SLAM algorithm for the dairy cow 3D reconstruction problem may encounter issues such as lost tracking during the point cloud registration process between fragments, leading to distorted 3D results and the emergence of numerous outliers.

**Figure 2:**
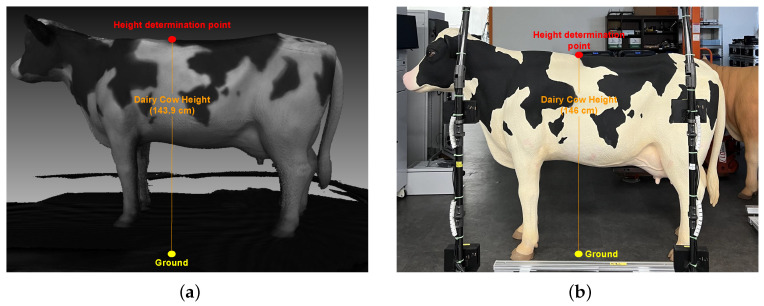
Dairy cow 3D reconstruction evaluation: (**a**) 3D dairy cow point cloud. (**b**) Dummy dairy cow.

To ensure the accuracy of the dairy cow 3D reconstruction system, an evaluation was conducted on a non-moving dairy cow model, as shown in Figure 2.

The error between the results measured manually on a dummy dairy cow and the results measured on a 3D reconstruction file is E=2.1 cm. According to the depth quality specifications provided by the Intel Realsense datasheet, RMSError≤ 4 cm for the objects is within 2 m and 80% ROI. For body length or other dimensions of the cow that are within approximately 1 m from the camera, the error is only around 1 cm. Therefore, our 3D reconstruction algorithm can create highly accurate 3D dairy cow objects used for auto-measurement problems.

### 3.1. The Camera Synchronization System

In the context of reconstructing a 3D object, achieving precise 3D geometric information poses a challenge when relying on a solitary camera. To enhance the capture of detailed geometric features in a 3D object, it becomes necessary to augment the number of cameras involved in the process. Nevertheless, the synchronization of these cameras is crucial for the simultaneous capture of frames. Incorrect synchronization among cameras can lead to the generation of numerous artifacts in the reconstructed 3D object. As presented in [9], the author has provided a comparison of the quality of 3D reconstructions created based on two different approaches to show the importance of camera synchronization. Our multi-camera synchronization system can synchronize 10 cameras together through one host (Jetson Orin).

**Figure 3:**
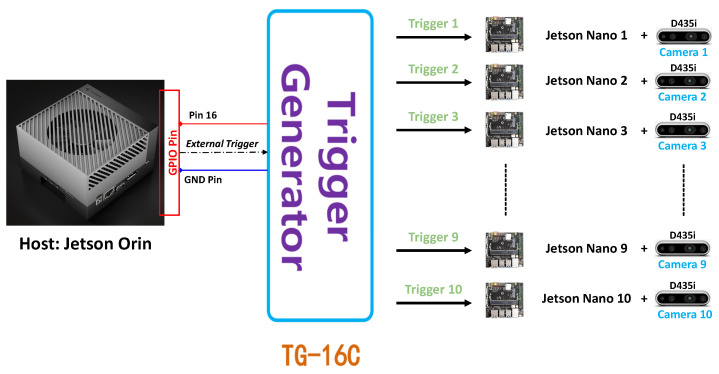
Genlock synchronization system.

In the current work, only 2 out of 10 pairs of Jetson Nano and Intel RealSense cameras are used to collect RGB-D data for the dairy cow 3D reconstruction problem. This system has been developed with the purpose of aligning the frames acquired between two cameras placed in the top and bottom positions. All frames were captured by cameras with corresponding timestamps, as shown in Figure 3. However, synchronously capturing frames via an external trigger signal proves challenging to ensure synchronization when storing frames from the RealSense camera to the host. Therefore, synchronizing the global timestamps among host computers with each other based on the Network Time Protocol (NTP) [12,13] has been applied to achieve the simultaneous computation of frames. Consequently, we can retrieve simultaneously captured frames by gathering each frame from all cameras that share the same global timestamps.

### 3.2. Dairy Cow 3D Reconstruction Improvement

**Figure 4:**
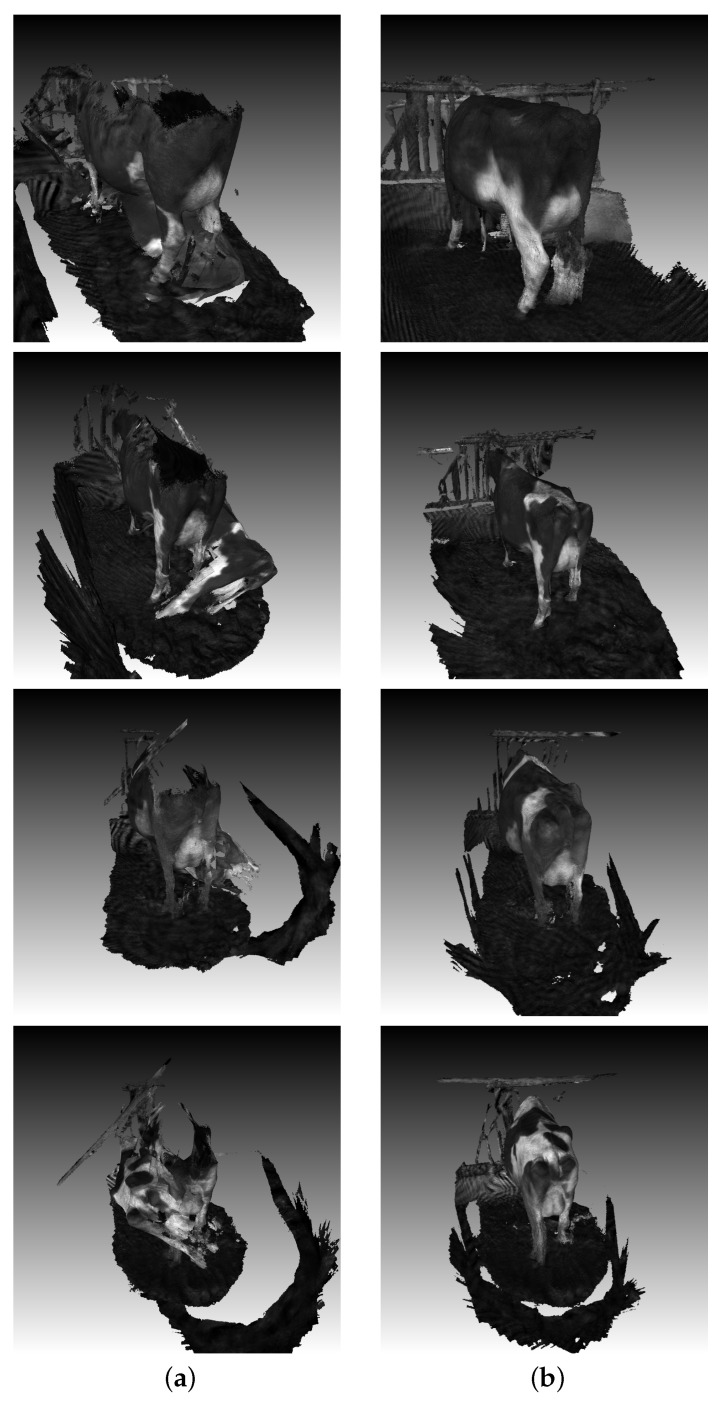
Dairy cow 3D reconstruction improvement: (**a**) Merged 3D dairy cow point cloud based on old algorithm. (**b**) Merged 3D dairy cow point cloud based on a new algorithm.

As presented in [14], dairy cow 3D reconstruction is generated based on the RGB-D dataset applying an AI algorithm for creating depth images. Initially, k-frame segments were derived from pre-existing short RGB-D sequences. Within each subsequence, the RGB-D odometry algorithm [15] was applied to ascertain the camera trajectory and merge image ranges. Specifically, the identity matrix served as the initialization for adjacent RGB-D frames. In contrast, for non-adjacent RGB-D frames, ORB feature computation facilitated sparse feature matching across wide baseline images [16], followed by a 5-point RANSAC [17] process for a preliminary alignment estimation, which was utilized as the initialization for RGB-D odometry computation. To determine the appropriate k value, considering the number of input frames, k = 100 was consistently set for all experiments with the current dataset. Utilizing the initial 100 frames, fragments were generated, providing a description of a segment of the dairy cow surface mesh. Once the fragments of the scene are generated, the next step involves aligning them in a global space. Global registration refers to an algorithm that operates without the need for an initialization alignment. Typically, it computes and offers a less rigid alignment, serving as the initialization for local methods such as the ICP (Iterative Closest Point). In the current work, we used FGR [18] to initialize alignment. However, the rate of creating full 3D dairy cow reconstruction is quite low after some experiments. As shown in Figure 4, it is not possible to stitch the point cloud created from the top camera (containing information mainly about the back of the dairy cow) with the point cloud created from the bottom camera (containing information mainly about the lower body of the dairy cow). Two parameters, Fitness and Inlier RMSE, are calculated to evaluate our algorithm, as shown specifically in Table 1. On the dataset of some dairy cows, we applied the algorithm for 3D point cloud registration and evaluated it. The Fitness value after updating the algorithm increased from 0.1 to 0.5, and the Inlier RMSE value also decreased from 0.02 to 0.01.

**Figure 5:**
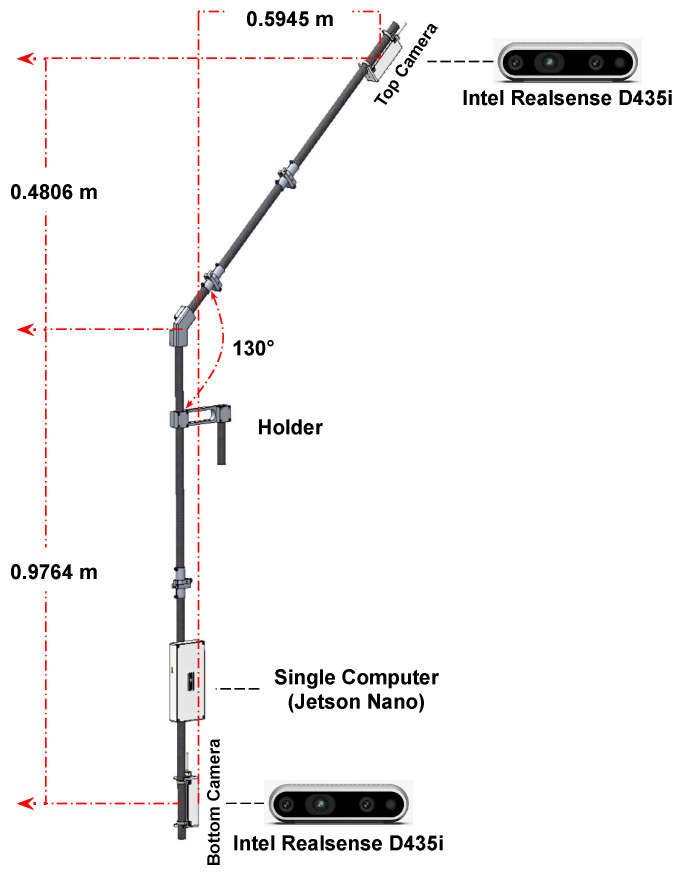
Dairy cow stereo dataset recording system.

Therefore, an initialization matrix T has been created based on the physical parameters of the camera’s position in the dairy cow data collection system, as shown in Figure 5. Equation (1) presents the specific values of the T matrix. By combining the FGR algorithm with these values, the point clouds generated from the top camera are rotated and translated closer to the nearest point cloud generated from the bottom camera.

Specifically,

(1) $$T=\left[\begin{array}{cccc} 0.642788 & -0 & 0.766044 & -1.39093\\ 0 & 1 & 0 & 0\\ -0.766044 & 0 & 0.642788 & 0.733545\\ 0 & 0 & 0 & 1 \end{array}\right]$$

### 3.3. Dairy Cow 3D Point Cloud Extraction and Normalization

**Figure 6:**
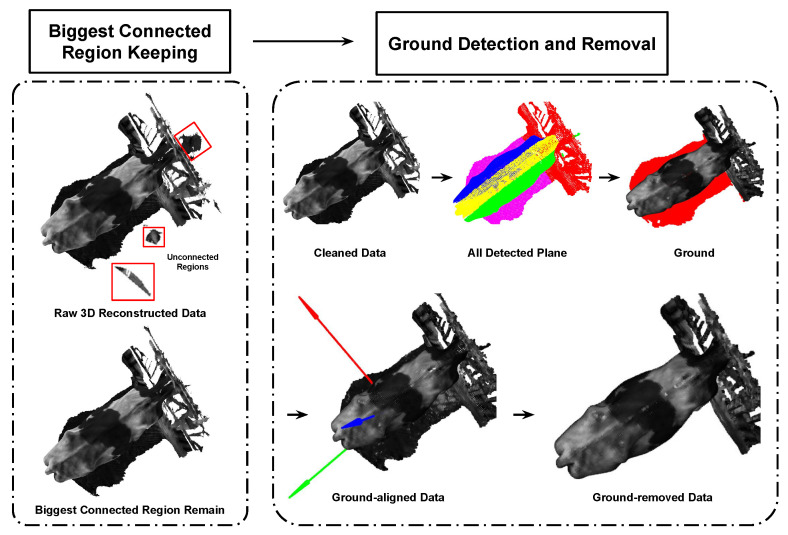
Dairy cow 3D point cloud extraction and normalization.

The 3D point clouds of dairy cows generated by the 3D reconstruction algorithms in [14] contain information from the input image scene. To expedite point cloud processing and mitigate the impact of the substantial volume of point cloud data on computer resources, the Voxel Grid approach was employed for downsampling the point clouds [19]. The original point cloud data include points from the target dairy cow, the fence, the ground, and outliers. To minimize the influence of unnecessary point clouds, the dairy cow clouds were trimmed at the plane of the fence and the ground. As depicted in Figure 6, the raw 3D reconstruction data encompass two main planes: the ground and the fence. The RANSAC algorithm was utilized to facilitate the detection of these two planes. This algorithm randomly selects three points within the point cloud, estimates the corresponding plane equation Equation (2), and utilizes the distance d to identify points belonging to the plane. Through multiple iterations, the plane with the greatest number of points was extracted.

(2) $$\begin{array}{c} ax+by+cz+d=0\\ n^{T}x=-d \end{array}$$

where,

$n={\left[\;n_{1}\;n_{2}\;n_{3}\;\right]}^{T}$: the normal vector of plane

$x={\left[\;x_{1}\;y_{1}\;z_{1}\;\right]}^{T}$

## 4. Automated Measurement and Analysis of Dairy Cows via 3D Point Cloud

### 4.1. Dairy Cow Body Automated Measurement

Considering the fragmented 3D shape of an animal, which is depicted as an amalgamation of point clouds acquired from a set of two depth cameras $\left\{P_{1},P_{2}\right\}$, the point clouds are concatenated into a unified single point cloud such as $P={⋃}_{c=1}^{2}P_{c}$, where $P=\left\{p_{1},\ldots ,p_{n}\right\}$ and $p_{i}\in R^{3}$. Our objective is to determine the m ordered key points $N=\left\{n_{1},\ldots ,n_{m}\right\}$, where $n_{j}\in P$. The key points are systematically annotated in a consistent order, such as right rear leg, right front leg, hip, …, giving them a well-defined semantic significance. The coordinate system is defined by the x, y, and z axes, respectively, which are displayed as red, green, and blue arrows, as shown in Figure 6.

As described in [20], the key points extraction was proposed as a regression problem. Specifically, the distance between each point in the point cloud and each annotated point was computed, yielding m distance vectors, each with a size of n. Utilizing these distances, the key points can be determined by identifying the point with the minimum value in each distance vector. From a machine learning perspective, the problem is formulated as a mapping between the n×3 input matrix P and the n×m output matrix $\hat{D}$. This naturally lends itself to an encoder–decoder architecture, where features for each point are aimed to be predicted from the input. Point cloud encoder–decoder architectures are typically designed to address semantic segmentation problems, where the probability of each point belonging to a specific class is predicted by the neural network. The transformation of an encoder–decoder into a segmentation problem is achieved by converting the network’s prediction into a probability, typically through the use of a sigmoid function, and by having the expected class probability backpropagated through the loss function. In this research, we contend that an encoder–decoder architecture can be employed interchangeably provided that it possesses the capacity to learn from point clouds [21].

The task of detecting specific points on a cow’s body is formulated as a 3D key point detection problem using point cloud data. To tackle this challenge, we leverage PointNet [22], a specialized 3D Deep Neural Network (DNN) designed for comprehensive 3D data analysis, offering the unique capability to learn both global and local features.

**Figure 7:**
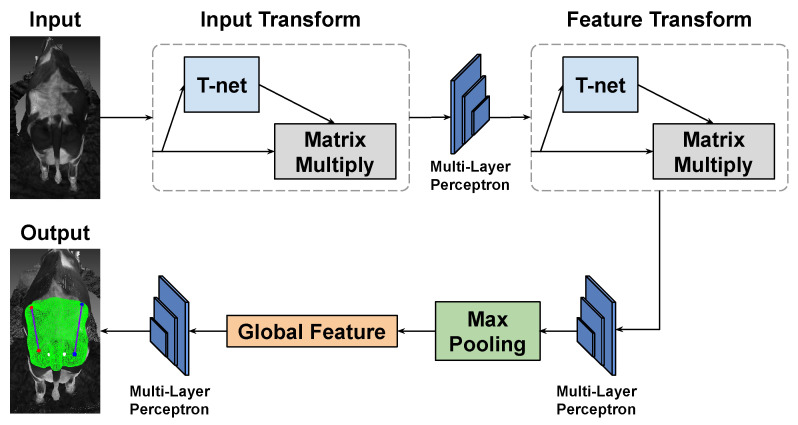
PointNet architecture for 3D dairy cow segmentation.

In Figure 7, the architecture of PointNet is structured as follows: it incorporates an Input Transform Network (T-Net) succeeded by a sequence of Multi-Layer Perceptrons (MLPs) dedicated to local feature extraction. The Input Transform Network adeptly captures transformations, ensuring the network’s resilience to variations in input point permutations, rotations, and translations. Following this, a Feature Transform Network (T-Net) is employed to augment the network’s ability to handle diverse point orderings. Upon local feature extraction, a global feature vector is derived through max pooling, facilitating the aggregation of information from the entire point cloud. This global feature vector undergoes further processing by a set of MLPs, culminating in the production of the final segmentation mask. This mask assigns class labels to each individual point, effectively completing the task. The synergistic interplay between the Input and feature Transform Networks empowers PointNet to robustly extract features from point cloud data, making it a potent solution for the nuanced task of detecting specific points on a cow’s body.

In this work, the model PointNet is implemented by Pytorch, which is a popular Deep Learning framework. Details of the training environment are as in Table 2.

As present in Table 3, dairy cow body part measurement values refer to quantified data associated with various physical characteristics and dimensions of dairy cows. These measurements play a crucial role in assessing the health, well-being, and productivity of the animals in a dairy farming context.

The following are explanations for some of these key measurement values:

***Stature Value:*** Stature value refers to the measurement of a dairy cow’s height, which is usually from the ground to a specific point on its body. This measurement is vital in determining the cow’s overall size and can be used to monitor growth, nutritional status, and assess the animal’s ability to access feed and water resources.

***Rump Angle Value:*** Rump angle value measures the angle of a cow’s rump, specifically the slope from its lower back to the tailhead. The rump angle can provide insights into the cow’s body condition and reproductive health. Changes in rump angle can indicate shifts in body fat and muscle distribution.

***Rump Width Value:*** Rump width value quantifies the width of the cow’s rump, which is the area just before the tailhead. This measurement can help assess the cow’s body condition, particularly the development of the pelvic area, which is essential for calving.

***Front Teat Length:*** The front teat length is the measurement of the length of the teats on the front udder of a dairy cow. This measurement is essential in assessing udder health and milkability. It can also be an indicator of the cow’s ability to nurse its calf or be milked efficiently.

These measurement values are collected through various methods, including manual measurements and automated systems that employ advanced technologies such as 3D point cloud imaging. Accurate and consistent measurements are essential for monitoring the health and performance of dairy cows, enabling farmers to make informed decisions about their care, nutrition, and overall management.

### 4.2. Stature Height

**Figure 8:**
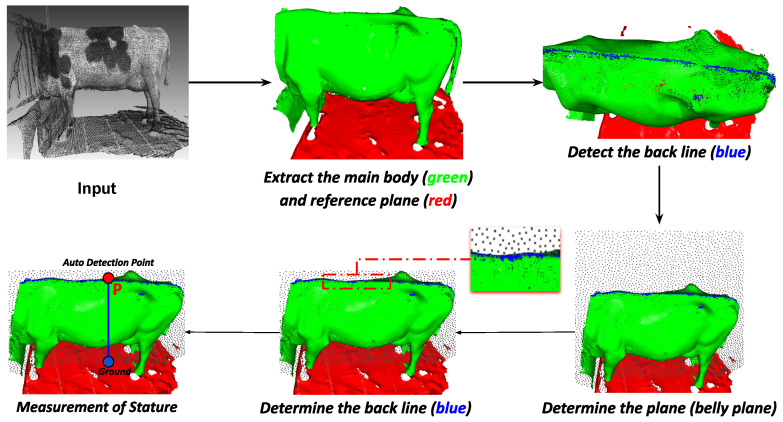
Measurement of stature height.

The stature height of a dairy cow is the vertical distance from the highest point on the back of a dairy cow to the ground. As illustrated in Figure 8, the 3D point cloud of the dairy cow after being separated from the ground plane is utilized for the computation of stature height. By applying depth learning techniques to identify key point clouds, the stature height can be calculated through point-to-plane distance measurements, $M_{StatureHeight}$.

(3) $$\begin{array}{c} M_{StatureHeight}=d\left(P,GroundPlane\right) \end{array}$$

### 4.3. Rump Angle

**Figure 9:**
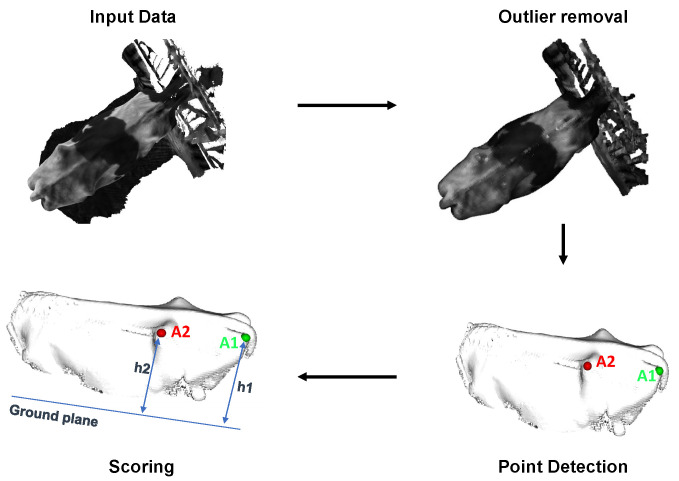
Measurement of rump angle.

The rump angle of the dairy cow is the inclination from the yaw angle to the sit bone. In Figure 9, it is shown that starting from the original dairy cow 3D point cloud, the outliers are removed to reduce computational costs and enhance the accuracy of key point detection based on AI.

Once the two key point clouds ($A_{1}$ and $A_{2}$) used to compute the rump angle are identified, the measurement is evaluated by comparing the distance between two points to the ground plane ($h_{1}$ and $h_{2}$) and providing a score. From the detected points $A_{1}$ and $A_{2}$, we can compute the distances from these points to the ground plane, $M_{RumpAngle}$

(4) $$\begin{array}{c} M_{RumpAngle}=h_{1}-h_{2} \end{array}$$

### 4.4. Rump Width

**Figure 10:**
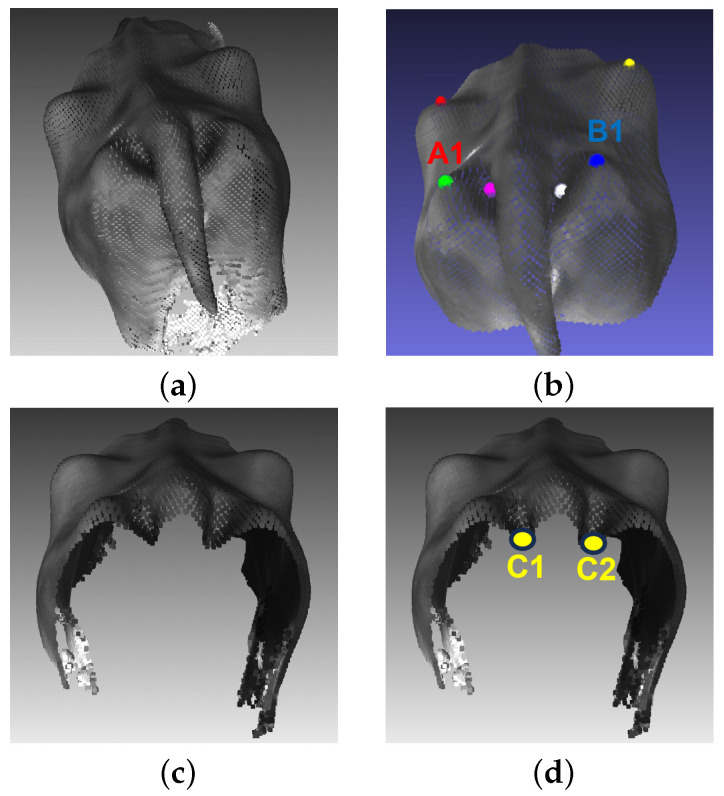
Measurement of rump width: (**a**) Input. (**b**) Detect points $A_{1}$ and $B_{1}$. (**c**) Cut body along point $A_{1}$, $B_{1}$. (**d**) Determine point $C_{1}$, $C_{2}$.

The rump width of a dairy cow is the inner width of the ischium and the width of the cone, $M_{RumpWidth}$. Figure 10 shows how to determine two points $C_{1}$, $C_{2}$ based on the AI algorithm for calculating rump width

(5) $$\begin{array}{c} M_{RumpWidth}=d\left(C_{1},C_{2}\right) \end{array}$$

### 4.5. Front Teat Length

**Figure 11:**
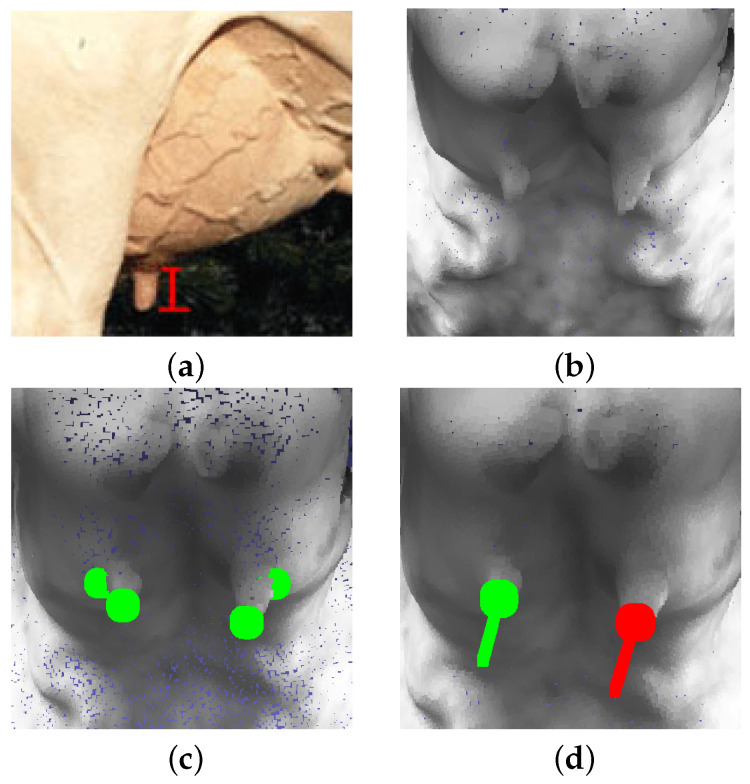
Measurement of front teat length: (**a**) Input. (**b**) Generated teat point cloud. (**c**) Teat labeling. (**d**) AI-based teat auto-detection.

After creating the 3D point cloud teat data (Figure 11b), this dataset is used to label for training and testing. Specifically, label 2 points at the top and the bottom of each teat (Figure 11c). With the automatic teat detection results after the training process (Figure 11d), the front teat length $M_{FrontTeatLenght}$ is determined according to Equation (6)

(6) $$\begin{array}{c} M_{FrontTeatLenght}=\frac{M_{left}+M_{right}}{2} \end{array}$$

where

$M_{left}$: the front teat length left

$M_{right}$: the front teat length right

## 5. Experimental Results

### 5.1. The Camera Synchronization System

**Figure 12:**
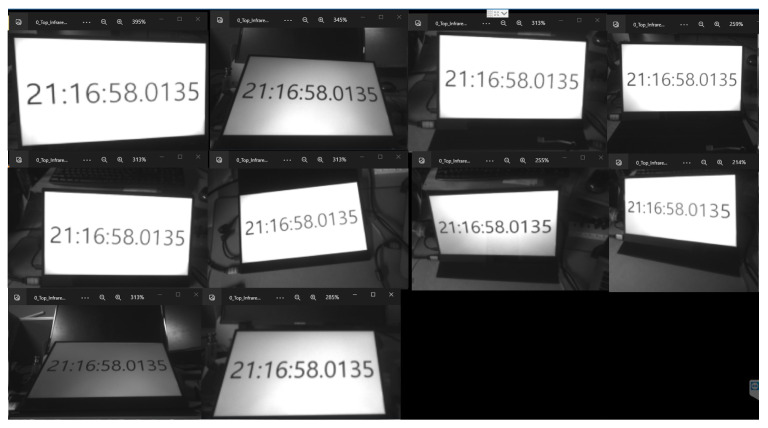
The synchronization results.

To assess the synchronization between the two cameras in the dairy cow 3D reconstruction system, we constructed a system comprising ten cameras connected to 10 Jetson Nano single computers, and all the data collected were sent to a host computer, Jetson Orin. The device specifications are detailed in Table 4. The synchronization was demonstrated through the frames obtained from the 10 cameras, which displayed the same timestamp on the screen when they had the same index. The synchronization results is shown in Figure 12.

### 5.2. Dairy Cow Body Automated Measurement

The key to calculating the body size data for cows, including withers height, body length, chest width, and chest girth, lies in the measurement points on the cow’s body. The automated cow body measurement algorithm was applied to the cow’s point clouds after coordinate normalization and refinement. The definitions for manual and automatic measurement values for each body size were as follows.

#### 5.2.1. Stature Height

**Figure 13:**
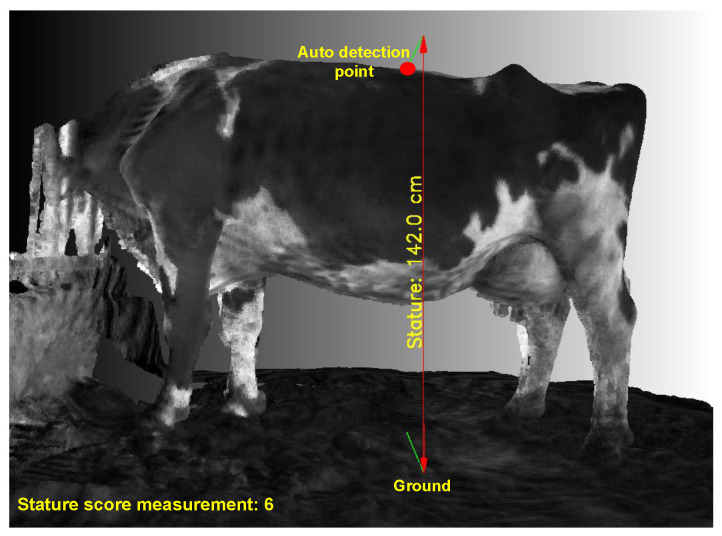
Auto-detection and measurement of stature height.

As shown in Figure 13, by adding the calculated value and score measurement directly to the input 3D point cloud dataset (statureheight = 142.0 cms and scoremeasurement = 6), the evaluating and monitoring of the condition of dairy cows becomes simpler and more intuitive for evaluators via any point cloud file (.ply) display application. Table 5 displays the outcomes of the non-contact measurement system’s repeatability.

The mean absolute error of 0.7 cm in height measurement indicates a relatively high level of precision in capturing the vertical dimension of dairy cows. This accuracy is crucial in assessing the growth, health, and overall stature of the animals. It can aid in determining the appropriate feeding and care for each cow within the herd.

#### 5.2.2. Rump Angle

**Figure 14:**
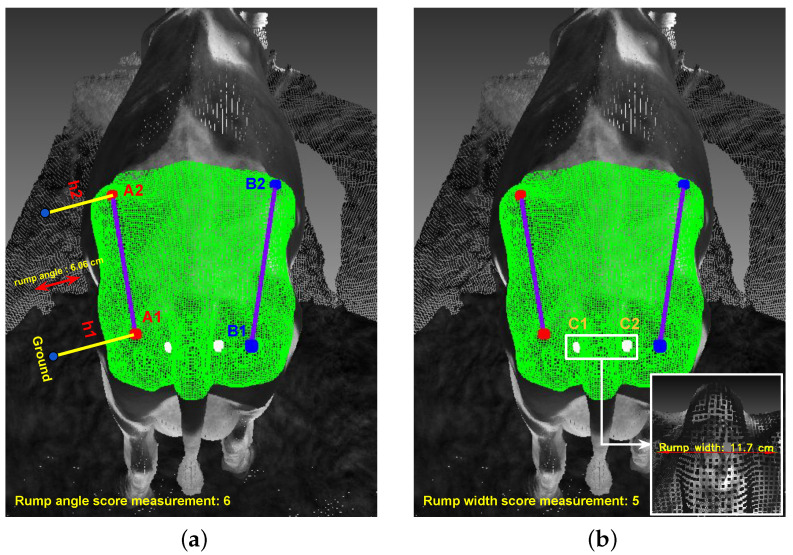
Auto-detection and measurement of rump part: (**a**) Rump angle. (**b**) Rump width.

Figure 14a shows that four points $A_{1},A_{2}$, and $B_{1},B_{2}$ are automatically determined through the AI algorithm for the rump angle measurement problem. They are then calculated and given a score measurement to display on the rump part point cloud (the green point cloud area), rumangle=6.06 and scoremeasurement=6.

The measurement of rump-angle auto-detection error on 101 samples is computed in Table 6. The 0.61 cm mean absolute error in rump angle measurement reflects the depth cameras’ competence in quantifying the inclination or tilt of the cow’s rump. This metric is valuable in assessing the cow’s comfort and posture, which are particularly relevant for dairy cattle’s well-being and milking efficiency.

#### 5.2.3. Rump Width

Similar to the rump angle, the rump width is calculated through two automatically determined points $C_{1},C_{2}$, as shown in Figure 14b. The calculated rump width value and score measurement are added directly to the 3D point cloud input. Specifically, rumpwidth = 11.7 cm corresponds to scoremeasurement=5. The auto-detection error of rump-width measurement on 101 samples is computed and shown in Table 7. With a mean absolute error of 2.5 cm, the depth cameras demonstrate their capability to accurately capture the width of the cow’s rump. This measurement is significant in evaluating the body condition and reproductive health of the animals. The precision achieved here contributes to the effective management of dairy herds.

#### 5.2.4. Front Teat Length

**Figure 15:**
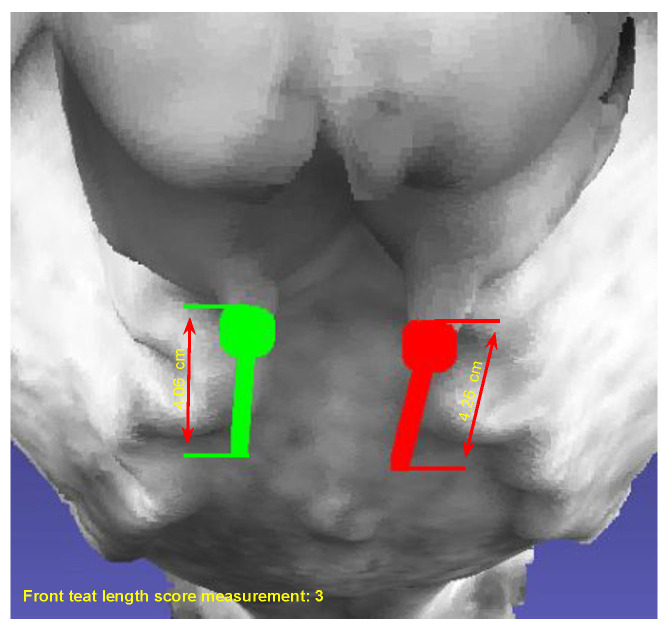
Auto-detection and measurement of front teat length.

After the dairy cow teat is automatically detected, the teat length value is calculated and given a score measurement as shown in Figure 15, teatleftlength=4.06, teatrightlength=4.26, averageteatlength=4.16 and scoremeasurement=3. Besides, the result was computed on 71 samples as shown in Table 8.

The front teat length measurement, with a mean absolute error of 0.79 cm, is important for the assessment of milking efficiency and udder health. This level of accuracy enables dairy farmers to make informed decisions about milking routines and cow comfort.

The error between the manually measured values of dairy cow height, rump angle, front teat length and values calculated by the auto-measurement algorithm were within 0.7 cm. The errors observed did not surpass those generated by manual measurements.

In the verification, the rump width has shown a large error compared to other body parameters, which is possibly due to the structure of the rump width calculation area being quite complicated and the two points to be detected being too close together, leading to confusion in determining the point to measure based on AI.

Table 9 presents an analysis of research conducted for non-contact body measurement applications. Specifically, the measurement method, type of devices used to collect processing objects, and type of data processing objects (2D images, depth images, and point cloud) are important criteria that affect the results the 3D point cloud produces as well as the accuracy in automated measurement and analysis of animals via a 3D point cloud. This table also presents some measuring objects on animals such as horses, cows, pigs, and dairy cattle along with the performance of different measurement methods.

Precision requirements in dairy farming statistics can vary based on the specific applications and objectives. Precision is crucial in ensuring the accuracy and reliability of data collected for various aspects of dairy farming. In our work, accurate measurements of cow height (stature) are crucial for monitoring health and productivity. The mean absolute error (MAE) of 0.7 cm is acceptable in many scenarios—especially if the primary goal is to identify significant changes rather than precise measurements. Precision in rump-angle measurement is critical for assessing cow body condition and comfort. Based on our current approach, the MAE of 0.61 cm is relatively low and should be considered satisfactory for most practical purposes in dairy farming. The rump width is a significant factor in determining the optimal space required for a cow to move comfortably. The MAE of 2.5 cm may be acceptable in certain applications, depending on the specific needs of the dairy farm. However, if precision is crucial for a particular task, further improvements may be considered. Especially, front teat length is an important parameter for milking efficiency and udder health. The MAE of 0.79 cm is generally acceptable for routine monitoring in dairy farming.

Finally, this research has contributed to practical significance in the field of automated measurement and analysis of animals via 3D point clouds. The low mean absolute errors in these body measurements highlight the feasibility of using depth cameras for accurate and non-invasive data collection in the dairy industry. This technology can significantly improve the management and well-being of dairy cows, leading to increased milk production, health, and overall farm efficiency. Additionally, the results from this study could contribute to precision livestock farming practices, enabling farmers to make data-driven decisions for their dairy herds.

## 6. Conclusions

In conclusion, this paper has presented a pioneering approach to revolutionize the measurement and analysis of dairy cows through the innovative use of 3D point cloud technology. The intersection of precision livestock farming and advanced sensing techniques has yielded a system that promises to redefine how dairy farmers monitor and manage their herds. Through a comprehensive review of related work, we have contextualized our contributions within the broader landscape of automated livestock monitoring, highlighting the unique advantages offered by 3D imaging and point cloud data. Our proposed system leverages 3D imaging sensors to generate high-resolution point clouds, capturing intricate details of dairy cow anatomical structures. The developed automated algorithms extract crucial metrics related to body dimensions, posture, and health indicators, providing farmers with a nuanced understanding of individual and herd-wide conditions. By automating these processes, our system not only alleviates the burden on farmers but also facilitates continuous, real-time monitoring, enabling early detection of health issues and timely interventions. Furthermore, the integration of machine learning algorithms enhances the system’s capability to identify and classify various behaviors and conditions, contributing to a more comprehensive assessment of dairy cows’ well-being. While the presented work marks a significant stride forward, we acknowledge that further research is needed to refine and expand the capabilities of our system. Additionally, collaboration with stakeholders, including farmers, veterinarians, and agricultural technology developers, is essential to ensure the practicality and adoption of our proposed approach in real-world dairy farming scenarios. In summary, our work contributes to the growing body of knowledge in precision livestock farming by introducing a methodology for the automated measurement and analysis of dairy cows. The fusion of 3D point cloud technology and machine learning algorithms represents a powerful synergy that holds promise for the future of dairy farming, where technology aligns with ethics and sustainability, fostering a new era of intelligent and humane livestock management.

## Figures and Tables

**Table 1:** Dairy cow 3D reconstruction evaluations.

Cow ID	Old Algorithm		New Algorithm	
	***Fitness***	***Inlier RMSE***	***Fitness***	***Inlier RMSE***
501363094	0.157	0.023	0.525	0.018
501363095	0.135	0.024	0.474	0.017
501208698	0.163	0.026	0.442	0.017
501349142	0.153	0.024	0.434	0.018

**Fitness:** the overlapping area of inlier correspondence set between source and target point cloud; the higher values are better. **Inlier RMSE**: the RMSE of all inlier correspondences metrics; the lower values are better.

**Table 2:** Details of the training environment.

Operation System	Windows 10
Python Version	3.8.17
Deep Learning Framework	Pytorch 1.13.1
Loss Function	Mean Squared Error
Optimization Algorithm	Adam [23]
Learning Rate	0.001
Number of Training Epochs	100 (without early stop)

**Table 3:** Dairy cow body part measurement value.

Stature Value (cm)	Rump Angle	Rump Width (cm)	Front Teat Length (cm)	Score Measurement
128 (very small)	The left hip is 4 cm above the iliac crest	5	2	1
131	The left hip is 2 cm above the iliac crest	6.5	3	2
134 (small)	The hip and iliac crest are level	8	4	3
137	The left hip is 2 cm below the iliac crest	9.5	5	4
140 (medium level)	The left hip is 4 cm below the iliac crest	11	6	5
143	The left hip is 6 cm below the iliac crest	12.5	7	6
146 (large)	The left hip is 8 cm below the iliac crest	14	8	7
149	The left hip is 10 cm below the iliac crest	15.5	9	8
152 (very large)	The left hip is 12 cm below the iliac crest	17	10	9

**Rump Angle**: Position of the left hip relative to the iliac crest (above, equal, below).

**Table 4:** The specification of hardware device.

Device	Specification
Depth Camera (Intel RealSense D435i)	- Use environment: Indoor/Outdoor - Baseline (mm): 50 - Resolution: 1920 × 1080 px - Frame rate: 30 fps - Sensor FOV (H × V × D): 69.4o × 42.5 × 77 (±3) - Dimensions: 90 × 25 × 25 mm - Connection: USB-C 3.1 Gen1
Single Board Computer (Jetson Nano)	- GPU: 128-Core Maxwell - CPU: Quad-core ARM Cortex-A57 CPU - RAM: 4 GB 64-bit LPDDR4 25.6 GB/s - Storage: microSD card slot for storage (256 GB) - USB: 4 × USB 3.0 ports, USB 2.0 Micro-B - Networking: Gigabit Ethernet - Wireless: Optional Wi-Fi/Bluetooth module - Operating System: Supports NVIDIA’s Linux-based operating system - Power: 5V/4A power supply
Host Computer (Jetson Orin)	- GPU: NVIDIA Ampere architecture with 2048 NVIDIA® CUDA® cores and 64 Tensor Cores - CPU: 12-core Arm® Cortex®-A78AE v8.2 64-bit, CPU - RAM: 64 GB 256-bit LPDDR5, 204.8 GB/s - Storage: 64 GB eMMC 5.1 storage - USB: Up to 2 × 8, 1 × 4, 2 × 1 (PCIe Gen4, Root Port and Endpoint), 3 × USB 3.2 - Networking: 1 × GbE, 1 × 10 GbE - Operating System: Supports various Linux distributions - Power: 15 W–60 W

**Table 5:** Stature measurement auto-detection error. The result was computed on 347 samples. Unit: cm.

Cow ID	Manual Height Measurement	Auto Height Measurement	Detection Error
500991129	145.28	144.87	0.41
501049585	149.09	147.79	1.31
501049591	147.21	146.78	0.43
501063723	142.87	142.27	0.60
501051848	151.02	150.54	0.48
⋮	⋮	⋮	⋮
501177073	148.97	148.29	0.69
501177573	148.34	147.70	0.64
501181588	147.17	147.32	0.15
501189051	146.92	147.02	0.09
501196133	148.03	147.90	0.13
**Detection Average Error**			**0.7**

**Table 6:** Rump-angle measurement auto-detection error. The result was computed on 101 samples. Unit: cm.

Cow ID	Manual Rump-Angle Measurement	Auto Rump-Angle Measurement	Detection Error
500991129	5.35	5.73	0.37
501034812	5.01	3.74	1.26
501049585	5.23	5.55	0.33
501049591	5.10	4.96	0.13
501051848	3.13	3.02	0.09
⋮	⋮	⋮	⋮
501324695	1.25	1.65	0.41
501324698	7.25	6.32	0.94
501324869	6.56	6.42	0.14
501326761	10.86	10.61	0.25
501326788	6.55	5.61	0.94
**Detection Average Error**			**0.61**

**Table 7:** Rump-width measurement auto-detection error. The result was computed on 101 samples. Unit: cm.

Cow ID	Manual Rump-Width Measurement	Auto Rump-Width Measurement	Detection Error
500991129	8.68	12.73	4.05
501034812	10.67	10.40	0.28
501049585	11.33	13.05	1.72
501049591	11.56	12.65	1.09
501051848	9.89	11.67	1.78
⋮	⋮	⋮	⋮
501324695	14.06	11.87	2.19
501324698	15.4	14.22	1.18
501324869	13.9	12.23	1.67
501326761	12.92	27.13	14.21
501326788	12.91	12.94	0.03
**Detection Average Error**			**2.5**

**Table 8:** Front teat length measurement auto-detection error. The result was computed on 71 samples. Unit: cm.

Cow ID	Manual Front Teat Length Measurement	Auto Front Teat Length Measurement	Detection Error
501031885	4.43	4.34	0.10
501049585	4.33	4.14	0.19
501093021	3.71	3.95	0.24
501105712	4.71	4.82	0.10
501118379	3.89	4.34	0.45
⋮	⋮	⋮	⋮
501381814	3.20	4.07	0.86
501382062	3.77	4.29	0.52
501382955	4.25	4.42	0.17
501383287	4.28	4.11	0.17
501386628	4.74	4.39	0.35
**Detection Average Error**			**0.79**

**Table 9:** An analysis of research conducted for non-contact body measurement applications.

Research	Measurement Method	Device	Object Processing	Animal	Object Measurement	Performance
Rodriguez Alvarez (2018) [7]	Automatic measurement	Kinect	Depth image	Cow	Body condition score	Accuracy: 78% within 0.25, Accuracy: 94% within 0.5
Nir et al. (2018) [2]	Automatic measurement	Kinect	Depth image	Cow	Hip height, withers height	Mean relative absolute error less than 1.17%
Zhang et al. (2019) [5]	Automatic measurement	Kinect	Depth image	Cow	Measurement points on the backside	Mean absolute error less than 1.17 cm
Weber et al. (2020) [1]	Automatic measurement	RGB camera	2D image	Cow	Feature points on the backside	N/A
Kuzuhara et al. (2015) [24]	Manual measurement	Xtion pro	Point cloud	Cow	Backside	N/A
Salau et al. (2017) [25]	Manual measurement	Six Kinect	Point cloud	Cow	Teat length, heights of the ischial tuberos	Standard error range are 0.7∼1.5 mm, and 14.0∼22.5 mm
Le Cozler et al. (2019) [26]	Manual measurement	Five LiDAR sensors	Point cloud	Cow	Volume and surface area	Coefficients of variation were 0.17% and 3.12%
Song et al. (2019) [27]	Automatic measurement	Three Kinect	Depth image	Cow	Vertebral column, centerline of the sacral ligament, hook bone center	N/A
Ruchay et al. (2020) [28]	Manual measurement	Three Kinect	Point cloud	Cattle	Withers height, hip height, chest depth, heart girth, ilium width, hip joint width, oblique body length, hip length, chest width	With a 90% confidence level, measurement errors less than 3%
Our	Automatic measurement	RGB camera	Point cloud	Dairy Cow	Height (stature), rump angle, rump width, front teat length	Height (stature): mean absolute error, 0.7 cm Rump Angle: mean absolute error, 0.61 cm Rump Width: mean absolute error, 2.5 cm Front teat length: mean absolute error, 0.79 cm

## Data Availability

Data are contained within the article.

## Abbreviations

The following abbreviations are used in this manuscript:

AI	Artificial Intelligence
NTP	Network Time Protocol
RANSAC	Random Sample Consensus
RMSE	Root Mean Square Error
ROI	Region of Interest
SLAM	Simultaneous Localization and Mapping
